# Challenges and opportunities in the implementation of a new robotic system – a semi-structured interview study with a surgical team

**DOI:** 10.1007/s11701-025-03097-4

**Published:** 2026-01-10

**Authors:** Ibrahim Alkatout, Désirée Meinhold-Heerlein, Paula Rosam, Henning Ohnesorge, Nicolai Maass, Sören von Otte, Rafał Watrowski, Thomas Becker, Julius Pochhammer, Claudia Bozzaro, Veronika Günther

**Affiliations:** 1 Department of Obstetrics and Gynecology, University Hospitals Schleswig-Holstein, Campus Kiel, Arnold-Heller-Straße 3 (House C), 24105 Kiel, Germany; 2 Department of Gynecology and Reproductive Medicine, University Hospitals Jena, Am Klinikum 1, 07747 Jena, Germany; 3 Department of Anesthesiology and Operative Intensive Medicine, University Hospitals Schleswig-Holstein, Campus Kiel, Arnold-Heller-Straße 3 (House R3), 24105 Kiel, Germany; 4 University Fertility Center, Ambulanzzentrum des UKSH gGmbH, Arnold-Heller-Straße 3 (House C), 24105 Kiel, Germany; 5 5Department of Obstetrics and Gynecology, Helios Hospital Müllheim, 79379 Müllheim, Germany; 6 Faculty of Medicine, University of Freiburg, 79106 Freiburg, Germany; 7 Department of General, Visceral, Thoracic, Transplantation and Pediatric Surgery, University Hospitals Schleswig-Holstein, Campus Kiel, Arnold-Heller-Straße 3 (House C), 24105 Kiel, Germany; 8 Institute of Ethics, History and Theory of Medicine, University Münster, Von-Esmarch-Straße 62, 48149 Münster, Germany

**Keywords:** Surgery, New robotic system, Interviews, Learning curve, Patient safety, Ethical issue

## Abstract

Introduction: Implementing a new robotic system poses a major challenge for the entire surgical team. We accompanied this sensitive initial phase of implementing a new surgical system in a clinic at which a previously established robotic system is used regularly. Materials and methods: Eight semi-structured face-to-face and video-based interviews were conducted with the surgical team involved in the implementation of a new robotic system (3 surgeons, 2 anesthesiologists, 2 operating room (OR) nurses and the head of OR management) between February and April 2025. Results: The new robot system finds its niche in bridging the gap between conventional laparoscopy and the established robotic system, allowing the advantages of the robot to be exploited in a wide range of operations. Although the established robot is the undisputed favorite for all interviewees, no ethical concerns regarding patient safety were expressed at any time. However, the wider range of applications and many years of experience with the established system seem to make it difficult to surpass it. Conclusions: The implementation of a new surgical system poses enormous challenges for the entire surgical team. Initial uncertainties regarding its handling can be overcome once the learning curve has been completed. A new surgical system is usually a technical and economic advancement that will benefit the clinic and patients, and thus the entire healthcare sector. In conclusion, new surgical systems should be given a chance, as they offer many advantages once the learning curve has been completed. Subsequently they contribute to scientific, economic, and clinical progress.

**Supplementary Information:**

The online version contains supplementary material available at 10.1007/s11701-025-03097-4.

## Introduction

Robotic surgery has become increasingly important in recent years, particularly in the fields of gynecology, general surgery and urology. The da Vinci robot still is the leading operative system. Following its market launch in 2000 by Intuitive Surgical, Inc., Sunnyvale, CA, USA, it has been the most frequently used robotic system throughout the world [1]. The expiry of the da Vinci patent gave rise to the introduction of other robotic systems in the market such as the Dexter Robotic System (Distalmotion SA, Epalinges, Switzerland), which was introduced in 2020 [2, 3].

The da Vinci robotic system has been in use since 2014, has successfully been established and is in the meantime considered to be on par with conventional laparoscopy. It has become an inseparable part of clinical routine and is no longer regarded as a major challenge for the surgical team. The learning curve has been completed and surgical procedures can be performed just as swiftly, with as few complications, as in conventional laparoscopy [4]. Similar to the surgeons who have completed the learning curve, the OR nurses, anesthesiologists, and surgical management are also accustomed to the new system; it has become an integral part of everyday clinical practice. In addition to the well-established da Vinci system, the Dexter robotic system is now to be implemented in clinical routine. The Dexter is a small, mobile platform designed to integrate into existing surgical environments, without the need for dedicated infrastructure. It consists of an open sterile surgical console, two patient carts, surgical units containing the robotic arms, and a separate endoscopic arm for imaging [5, 6]. Table 1 gives an overview concerning the differences between the new and the legacy robotic system. The Dexter robot was chosen as opposed to all of the other newly certified systems (Hinotori, Hugo, etc.) because the new system is closer to conservative laparoscopy and essentially has the advantages of robotics that the surgeons are familiar with. Furthermore, there is the desire to offer a financially different model as an alternative product. Handling and working with the Dexter robot are in the initial stage. The entire surgical team is embarking on the learning curve. Before starting with the first cases there was a mandatory curriculum on the virtual trainer as well as instructions in the operating room on the pelvic trainer. In addition, a manufacturer of Distalmotion was on site for every single operation in all specialist areas.

**Table 1:** Differences between the new (Dexter) and the legacy (da Vinci) robotic system

Feature	da Vinci System	Dexter System
**Modularity**	Dedicated, closed system	Modular and open-platform design
**Integration**	Requires proprietary equipment from the supplier	Seamlessly integrates with existing laparoscopic towers and equipment (e.g., 3D optics)
**Switching Modes**	More complex setup, including “docking”, which can take longer	Quick and easy transition between robotic and manual laparoscopic modes (15–30 s)
**Workflow**	Requires significant changes to the operating room setup and workflow	Adapts to existing surgical workflows, reducing the barrier to entry
**Instruments**	• Primarily uses proprietary robotic instruments • 4 robotic arms: one camera arm and three instrument arms	• Works with standard laparoscopic instruments and also has single-use instruments • 3 robotic arms: one camera arm and two instrument arms
**Space requirements**	Larger and requires significant operating room space	More compact and flexible for various operating room sizes
**Surgeon Console**	Closed console, which can make communication with the team more difficult	Open and sterile, allowing for easier communication with the surgical team

Clinical data and outcome are essential to justify the routine use of a new surgical approach. Nevertheless, this does not sufficiently explain the holistic challenge a hospital faces when introducing a new additional system. The transparency and clarification of a surgical challenge for the clinic and the surgical team will be addressed and discussed in the present study. The preliminary phase was accompanied and ethical concerns were addressed in implementing a robotic system that is basically established but still completely new to the surgical team. The latter, consisting of the surgeon, anesthesiologist, operating-room (OR) nurse, and surgical management, that accompanied the implementation phase of the Dexter robot, was interviewed for the present study. The interviews were focused on identifying the opportunities offered by the new robot-assisted system (advantages, communication, potential, and prospects). Furthermore, risks and concerns on the part of the surgical team are discussed. Is it ethically acceptable to use a system on patients that has not yet completed its learning curve, when a well-established robotic system is available that can be used to perform operations with virtually no error and a high degree of routine? Does the patient need to be informed about the new robotic system? Should the patient be able to choose?

We lack any legal requirements or guidelines that prioritize patient safety. A significant portion of the learning curve is traversed during the treatment of patients and this raises ethical concerns because a new technique signifies a learning curve and raises the following questions: does a new technique needs ethical approval and how would this be achieved [7]?

On the other hand, we are faced with scientific and economic progress: only by leaving the familiar and the “proven” will it be possible to achieve improvement, whether on a medical or economic level. This interview study addresses precisely this conflict: the desire for progress on the one hand and ethical concerns on the other. Does the desire for progress justify ethical concerns regarding patient safety? This study is not intended to be a direct comparison between two robot systems. Rather, it aims to discuss the fundamental situation in which a new system is introduced when there already is an existing one whose learning curve has been completed.

## Materials and methods

### Participants

Face-to-face and video-based semi-structured interviews were performed between February and May 2025, and eight individuals were interviewed who were all directly involved in the implementation of the Dexter system. Specifically, the interviewees consisted of three surgeons (two gynecologists and one general surgeon), two anesthesiologists, the head of surgical management, and two operating-room (OR) nurses. First, the interviewees were asked general personal questions, such as their age, specialization and professional experience. The average age was 41.9 ± 12.1 years and the average professional experience 16.2 ± 9.8 years. Table 2 summarizes these data [8].

**Table 2:** Participants`characteristics

Participant No.	Gender	Age (years)	Specialization	Medical function in the clinic	Professional Experience (years)
1	Female	49	Gynecology	Senior physician	22
2	Male	46	Gynecology	Senior physician	19
3	Male	48	General surgery	Senior physician	20
4	Male	59	Anesthesiology	Senior physician	31
5	Female	27	Anesthesiology	Assistant doctor	1
6	Male	49	Operating room management	Head of operating room management	21
7	Female	29	Operating room nurse	Operating room nurse	9
8	Female	28	Operating room nurse	Operating room nurse	7

### Interviews

The interview focused specifically on the Dexter robot, its implementation, and above all, a comparison of this new robot system and the well-established da Vinci robot, addressing topics such as communication in the operating room, safety from the surgeons’ perspective, and handling the new system (such as positioning the patient and docking). In addition, the participants were asked about economic motives (comparison of operating times) and ethical aspects (threat to patient safety, preference for a robotic system if, for example, a relative were to undergo surgery, comparison of error rates/incidents and the general atmosphere in the operating room, depending on the robotic system used).

The individual questions varied depending on the function of the interviewee. The anesthesiologists were asked especially about differences in positioning the patient, access to the patient, induction, and anesthesia in general with reference to the different robotic systems. The OR nurses and the operation room management were interviewed more on the topic of preparation and setup in relation to the different robotic systems and differences in transporting the two systems from one operating room to another. Finally, the surgeons were asked about their sense of safety and stress levels during surgery, possible differences in the duration of surgery, intraoperative error rates, and the possible influence of economic constraints on medical decisions. Finally, all interviewees were given the opportunity to comment on whether any topics had not yet been addressed (a), what kind of robot they would like to have if they could choose (b), and whether they had any other comments (c). The three different interview questionnaires can be found in the appendix.

All interview questions were developed jointly by I.A. and V.G. and in close consultation with the professor and head of the Institute for ethics, theory and history of medicine (C.B.).

### Data analysis

The interview transcripts were evaluated using qualitative content analysis based on Philipp Mayring’s approach [9]. This is based on a coding evaluation method with the aim of assigning categories to the data material and thus deciphering the research question posed at the outset. Mayring’s method combines “deductive category application” and “inductive category formation.” Deductive category application originates from the research question and theoretically defined definitions, thus forming the main categories. These are created after the first reading of the transcripts. Inductive category formation describes the creation of subcategories: aspects of evaluation are developed from the material [10].

The interviews were recorded with the prior consent of the participants and then transcribed using the special transcription company’s service *Amberscript*. The transcripts were then analyzed using the MAXQDA (version 24, VERBI GmbH, Berlin, Germany) software. This program offers the possibility of conducting a focused content analysis of the interviews by coding the interviewees’ statements into categories [11]. MAXQDA evaluates texts quantitatively by analyzing code frequencies, word statistics (such as type-token ratio), sentiment analysis, and descriptive statistics using the MAXDictio module and the Stats module to identify patterns and correlations and to present and compare results in tables (e.g., cross tables), diagrams (histograms, box plots), and statistical reports. The evaluation is carried out by converting qualitative codes into countable units and then statistically evaluating them, which enables a quantitative interpretation of the qualitative data. During the first round, the interviews were coded into their main categories (opportunities, risks/concerns). In the second step, sub-codings were then made, which enabled a more precise classification. The respective definitions of the codes could be recorded in so-called “memos.” The program also offered the option of recording initial thoughts and conclusions that arose during coding in the form of further memos or paraphrases. These initial thoughts could be included in the analysis at a later stage. After classification into categories, lists of the coded segments could be activated and displayed using a MAXQDA24 tool; these could then be used to record the results of the coded interviews.

The ethics committee of the Medical Faculty of Christian-Albrechts University of Kiel (Arnold-Heller-Str., House no. 9, 24105 Kiel, Germany) approved the study (Vote no. D 525/22, 18. August 2022). Informed consent was obtained from all participants before their inclusion in the study.

## Results

Eight participants were interviewed for the qualitative interviews. The interviewees consisted of surgeons, anesthesiologists, OR nurses, and operation room management, representing each professional group of the entire surgical team that was involved in the implementation of the new robot system. The written interview content was retrospectively evaluated in categories. The main themes were: opportunities, risks and concerns, advantages of the established system, and preferred system. These were in turn subdivided into subthemes. Table 3 gives an overview of the themes and subthemes and how often each category was mentioned. Some quotes were assigned exclusively to a main theme (but not to a subtheme), so that the sum of the subcategories does not always correspond to the total number of citations in the main category.

**Table 3:** Themes and subthemes that emerged from the interviews with the surgical team

Themes	Subthemes	Number of mentions/quotes (*n*)
Opportunities	• Advantages of the new system ▪ Functional advantages ▪ Adaptation • Communication • Potential and prospects ▪ Scientific exploration ▪ Economic progress	39 (total) 18 21 13 18 (total) 6 9
Risks and concerns	• Ethical conflicts ▪ Ethical acceptability ▪ Patient safety ▪ Economic interests • Acceptance of the new technology • Perioperative complications	77 (total) 18 26 33 12 18
Advantages of the established system		23
Preferred system		34

### Advantages of the new system

The subtheme “Advantages of the new system” was further divided into “Functional advantages” and “Adaptation.”

### Functional advantages

The participants primarily cited docking and undocking as functional advantages of the Dexter robot, noting that this proved to be faster with Dexter than with da Vinci, especially in less complex procedures. In this regard, the quick transition from the robot to the immediate action on the patient, as well as the aspect of open console architecture, was repeatedly highlighted as advantageous. Other functional advantages of the new robot included aspects of sterility and flexibility. From the perspective of the surgical team, it proved advantageous that the surgeon, wearing sterile clothing, could work directly on the patient at any time during the operation and thus be able to act quickly, especially in emergency situations: “*With Dexter it’s much faster because you*,* as the surgeon*,* can go to the table yourself. And you can help. And accordingly*,* even in situations where you have to undock or change something*,* the person at the table*,* i.e. the assistant*,* doesn’t need to have as much experience because you can always do it yourself.*”

The surgeon quoted here pointed out an aspect of autonomy for surgeons. With the Dexter robot system, the surgeon would theoretically no longer be dependent on the assistant’s support, as he or she could switch between the robot console and the patient table. This criterion was also recognized by other interviewees as a positive attribute of Dexter. In combination with a shorter operating time (surgeon: “*If we’re talking about comparable*,* uncomplicated procedures*,* then the operating time with Dexter is shorter because the docking time is shorter*”), this could also be profitable from the economic perspective.

### Adaptation

The subcode “Adaptation” covers the modifications that the system underwent, for example through updates. As part of the implementation of Dexter, these adjustments led to a learning curve, which in turn contributed to measurable success experiences for the surgical team. A total of 20 quotes were coded with the subcode “Adaptation.”

For the interviewees, the factors of time and training were essentially decisive for the successful establishment of the new system. All participants unanimously emphasized that, in their experience, it was essential to perform a large number of procedures with the new system in order to recognize its advantages and be able to make a comparable evaluation of both systems. A corresponding learning curve could be traced with the Dexter system:

*“And after we surgeons had overcome the learning curve*,* but also thanks to further development of the system*,* it has changed to such an extent that the Dexter system now really represents an improvement in many areas of surgery. But now*,* compared to robot-assisted surgery with the da Vinci system*,* the difference is still very significant.”*

Despite a noticeable learning curve, the cost-benefit ratio of the Dexter was viewed critically: “*But it didn’t establish itself as hoped. […] I think this is because the difference compared to laparoscopy is simply not big enough to justify the additional effort and costs.*”

### Communication

**Fig. 1:**
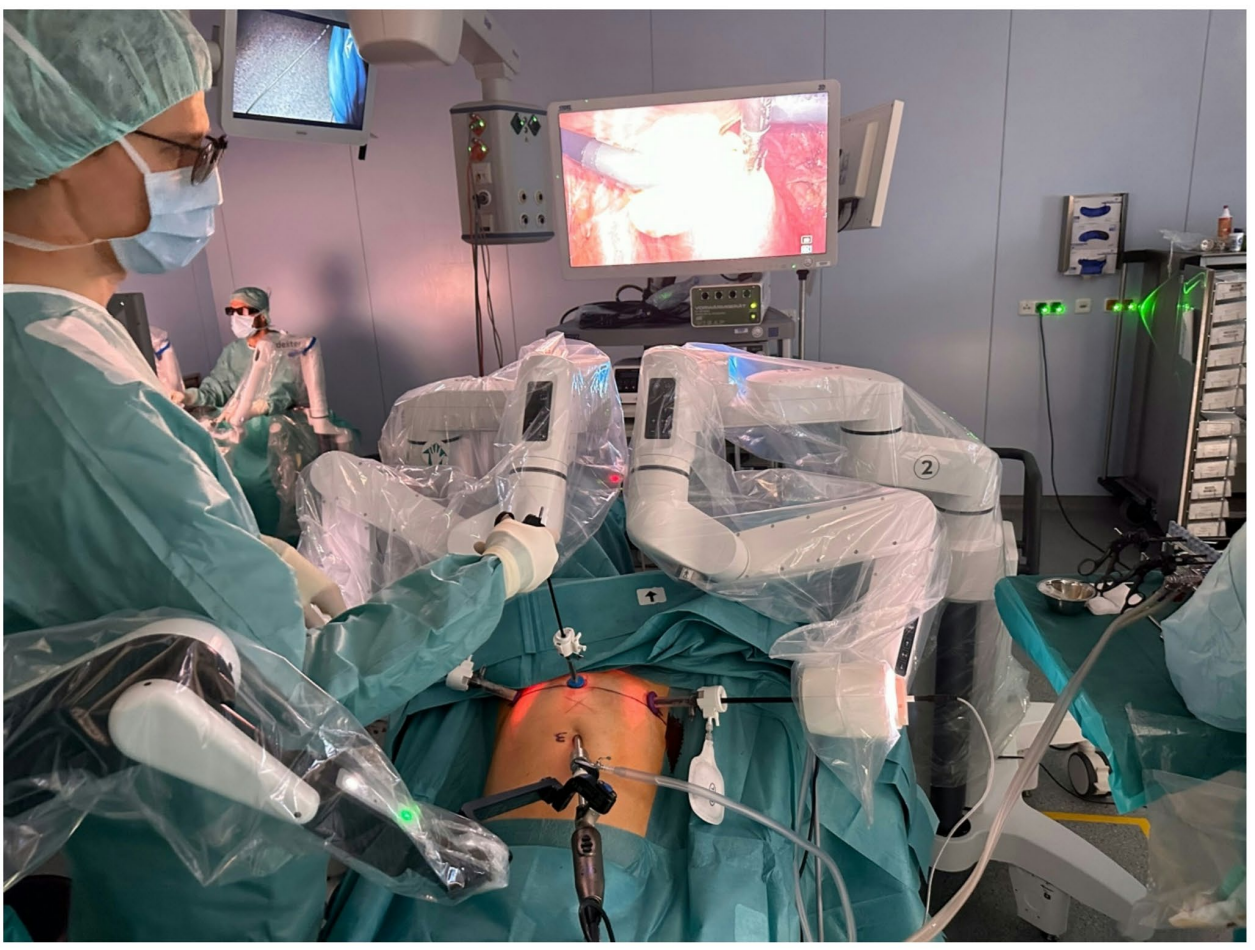
Intraoperative interactions within the surgical team. The Dexter system has an open console that improves the communication.

Another advantage of the new robot could lie in the area of improved communication. The subcode “communication” comprises 13 quotes. These highlight a possible optimization of intraoperative interactions within the surgical team, as the Dexter system has an open console (Fig. 1). In this context, the interviewees expressed shortcomings with regard to the da Vinci robot: “*The speaker quality and the communication quality is already limited. And if you have international nursing staff at the table*,* for example*,* who don’t speak German very well yet*,* that can be a problem. I’m a little surprised that the audio technology installed there is so mediocre.*”

Unlike the OR nurses, surgeons, and surgical management, the anesthesiologists did not perceive any difference in communication between the two systems. In contrast, both the OR nurses and the surgeons rated the nonverbal communication permitted by Dexter as being very advantageous.

### Potential and prospects

The interviews also addressed the potential and prospects associated with implementing a new system in the healthcare sector. In the 18 quotes for this subcode, the interviewees repeatedly discussed areas such as the teaching aspect, scientific exploration, and economic progress with a new system. Surgeons in particular believed that robot-assisted surgery will be the leading procedure in the future. For this reason, it is also crucial in terms of patient outcomes to implement robotic surgery in the best possible way. The interviewees emphasized the need for subsidies to enable such scientific progress and also praised the support and freedom of decision-making afforded by their hospital, which provided scope for scientific development: “*And ultimately*,* we are fortunate that this hospital has made a commitment to advocate innovative surgical concepts*,* even if they come at a higher price. This*,* in turn*,* gives us the freedom to make decisions in the best interests of our patients.*”

A new system of this type also offers great potential for scientific exploration. All interviewees shared the view that expertise will grow with increasing numbers of procedures, thus contributing significantly to scientific progress. Our working group has published 44 papers on the topics of laparoscopy, robot-assisted surgery, and their teaching and training.

When asked about their wishes or ideas for future robotic systems, the respondents expressed the idea of a certified center for robotic surgery: “*And [you] have a building where you can do these different things [robot-assisted*,* laparoscopic training]. And […] where you can offer different courses throughout the year*,* perhaps in collaboration with anatomy*,* with body donors.*”

In addition, interest was expressed in increased AI-based innovations: “*Then I would also like to see modern AI elements incorporated into the system to make the operation even safer. Similar to navigation*,* this would lead to guided surgery*,* which would further enhance patient safety and wellbeing. Those would be my next wishes.*”

The respondents said that the established da Vinci robot offered a good cost-benefit ratio, but was still considered very expensive. In their opinion, Dexter could not be a direct competitor to da Vinci, but could serve as a suitable additional system: “*I would say that da Vinci*,* which is simply the most expensive system*,* but also the most advanced*,* is the leading system for highly complex operations and interdisciplinary use. In terms of the cost-benefit ratio*,* however*,* there is certainly a niche for less advanced robotic systems*,* which then only cover a smaller spectrum but may be more cost-effective. The Dexter is ideal for this niche: it bridges the gap between conventional laparoscopy and the da Vinci system*,* allowing the advantages of the robot to be exploited in a wide range of operations*,* providing uncomplicated docking and undocking*,* and thus minimizing time loss.”*

### Risks and concerns

The implementation of a new technical system is always accompanied by obstacles and challenges. These risks and concerns, which arose during the establishment of the Dexter robot, are outlined in the following section. A total of 118 coded segments were summarized in this section (Table 3).

### Ethical conflicts

The codes under this heading may be divided into three subcodes: patient safety, ethical acceptability, and economic interests. In summary, 77 coded segments were assigned to the theme “Ethical conflicts.”

When implementing a new medical device, the focus is on patient safety and whether this can be guaranteed with the new system. The interviewees were questioned with this aspect in mind. Although the respondents confirmed their increasing familiarity with the Dexter, they perceived the surgeon’s handling of the da Vinci robot as safer: “*Yes*,* I would say it has improved*,* but you don’t have the absolute certainty that you had with the da Vinci. […] It was always a bit of a struggle*,* I would almost say. It wasn’t as smooth as with the da Vinci. The work wasn’t fluid. Everything was always sluggish and everyone was always a bit uncertain.”*

The respondents also shared their impressions of the atmosphere in the operating room. Three of the eight respondents felt that the overall mood was more stressful when operating with the Dexter. The surgical nursing staff attributed the tenser atmosphere to a lack of experience on the one hand and time pressure on the other, which was associated with greater susceptibility to errors. Rather than stress, the surgeons spoke of a temporary frustration when dealing with the Dexter. One surgeon described the state of mind as “*annoyed*,* but not stressed.*” Both the anesthetists and the surgical management found the atmosphere in the operating room similar with the two robotic systems.

All four groups perceived the patients as being equally calm preoperatively, regardless of the robotic system used to operate on them.

Finally, the interviewees were confronted with ethical questions. All interviewees answered the following question: “Do you see the patient’s wellbeing as being seriously endangered when operating with the Dexter?” Eight of eight interviewees gave a negative answer. They expressed the following views with regard to ethical conflicts:

1) “*I think it is justified to say*,* under the mentioned conditions*,* that we have limited access to the best possible system. Every day we have to make decisions about how to allocate the resources available to us in a sensible way. And all the forms we use in operating systems are approved systems*,* meaning they are established and approved systems in the market. They have passed the test for being included in an approval concept. In other words*,* in general there is nothing experimental about them.”*

2) “*It is a secure system in terms of its range of applications.*”

The introduction of a new medical device also raises economic questions and issues. According to the surgical management team, a shorter duration of surgery would save costs for the hospital: “*So*,* if there were [shorter surgery times]*,* there would be a cost saving. As a rule*,* robotic surgery saves little time. If anything*,* it tends to take longer.*”

Particularly at the beginning of the implementation of the Dexter robot, the respondents reported operating times that were sometimes twice as long than with the da Vinci or conventional laparoscopy.

Nevertheless, as the system became established, a learning curve and an improvement in terms of shorter operating times were observed. According to the interviewees, in order to achieve clear economic progress with a robotic system, only a system that “*allows us to fit in an additional surgical procedure during working hours*” would be useful. In addition to the initially longer duration of the operation, the additional consumables required and thus the ecological factor were also rated negatively. “*The cost per operation is simply too high*,* considering that I actually see relatively little improvement compared to conventional laparoscopy. The costs are also high with da Vinci*,* but I see a significant improvement.”*

### Acceptance of the new technology

An unbiased approach on the part of users is crucial for the successful establishment of a new medical device. The OR nurses were rather critical of the new system and did not see it as offering any significant added value: “*It just took up so much time. And especially for the older colleagues*,* it was simply a big change. […] It’s difficult to get everyone on board and to get people to even engage with it.*”

The surgeons expressed curiosity and anticipation about the new system, but were also aware of possible bias due to the established da Vinci robot: “*But I think that always depends on the individual. […] How you connect with this system and accept it. And anticipate it.*”

### Perioperative complications

During implementation, the surgical team reported several intraoperative technical problems such as error messages, especially at the beginning. The surgeons repeatedly cited incorrect power application as a source of error due to confusion between the pedals. Nevertheless, a positive development was also observed here with newer versions during the establishment phase.

### Advantages of the established system

The advantages of the da Vinci robot were summarized in 23 coded segments. In particular, the respondents rated the existing high level of expertise of the entire surgical team with the established da Vinci system as an advantage: *“Most of us are more familiar with the da Vinci system because we have been working with the first intuitive system since 2013. And that*,* of course*,* gives us a level of expertise that cannot be easily matched.*” Furthermore, the more complex procedures were largely performed with the established system. Four of the eight interviewees also favored the da Vinci because of its simpler docking and better handling. In particular, the fewer intermediate steps, greater reach, improved ergonomic physical maintenance, and absence of tremor were rated positively by the respondents. The absence of tremor also resulted in better image quality. Another beneficial aspect mentioned by the interviewees was the improved teaching concept at da Vinci. The dual console allows young surgeons to be trained more confidently in robot-assisted surgery: “*I believe that da Vinci is also very well suited for beginners. We have quickly trained many of our assistant doctors to use the da Vinci because we have these consoles where one person can essentially work alongside another.”*

The interviewees also rated the functional advantages of the established system as positive. These included the advanced energy devices and the additional third arm, which permits surgery without assistance.

### Preferred system

The code “preferred system” was assigned to 34 quotes. These included quotes in which the respondents expressed their preferences regarding one of the two robot systems. All interviewees answered the following questions:

1) Which robot do you generally consider the better one?

2) Would you give preference to one of the two robots if you yourself or a friend of yours were to be operated on?

All interviewees gave preference to the da Vinci for both questions. The cited reasons were greater experience and expertise, partly due to more frequent use. The wide range of applications of the da Vinci were also mentioned:

1) “*Because the EndoWrist instruments of the da Vinci offer greater flexibility and cover a wider range of surgical procedures. And thus simply offer a larger portfolio.*”

2) “*Yes*,* yes*,* I would choose the da Vinci*,* simply because it has been on the market longer. Because it is more established and because the surgeons are better trained to use it.*”

All interviewees believed that the Dexter will not be able to match the da Vinci in terms of use in the future. The interviewees considered the da Vinci’s lead to be unassailable:

1) “*The qualitative advantage of the da Vinci system is so much better than the Dexter system that I don’t believe this advantage can be overcome in the foreseeable future. Let’s take two examples: freedom of movement and restriction of movement. This is something that is extremely different*,* to the disadvantage of the Dexter system.*”

2) “*I wouldn’t say they are equivalent. I simply believe that the da Vinci is somehow the Porsche among robots.*”

## Discussion

In the present report, surgeons, anesthesiologists, OR nurses, and operation room managers were interviewed in order to evaluate the delicate phase of implementing a new robotic system. The interviewees were deliberately selected because they represent a cross-section of all members of the surgical team who were directly involved in the implementation. This study is not intended to be a direct comparison between two robot systems or an evaluation of their advantages and disadvantages to determine which is better. Rather, it aims to discuss the fundamental situation in which a new system is introduced when there is a previously existing system whose learning curve has been completed by all those involved. Is it really necessary and justified to introduce a new system when an existing one has already been integrated into everyday life and everyone involved is familiar with it and well-practiced in its use?

**Fig. 2:**
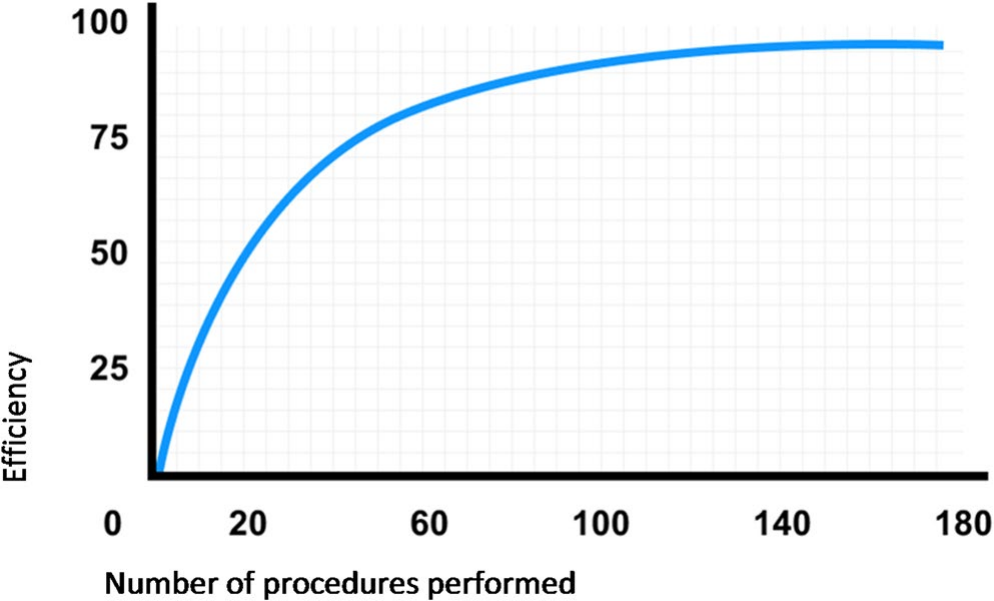
Learning curve. Relationship between a learner’s performance on a task and the number of attempts or time required to complete the task.

One potential advantage of a new system in general is the time factor, i.e., the respective surgical procedure can be performed more quickly, allowing more procedures to be completed overall, which would signify an enormous economic gain for the clinic. In the current report, this time factor was described as a positive one for the Dexter robot because docking and undocking were significantly less time-consuming than with the da Vinci system. However, recognizing this advantage requires a certain amount of experience and a learning curve in using the new system. The classic learning curve starts off steep and then flattens out over time, which can be explained by the increase in experience gained over time and improved skills (Fig. 2) [12].

A learning curve effect is defined as the improvement in surgical performance over time. At the beginning, the success of a new surgical method is quite often inferior to that of the standard method, which has usually been in use for a long time and has reached an asymptote in terms of learning success and cannot be further improved [13, 14]. Accordingly, the expected advantages of a new method only become apparent after a certain delay before the established procedures can, in fact, be surpassed [15]. When the da Vinci was first introduced in 2014 as the first robotic system to supplement conventional laparoscopy, significantly longer operating times were initially recorded which were due to a lack of routine in handling the system and difficulties with docking. In the meantime, the procedures have become so well-established and well-coordinated that there is no longer any difference in operating times for more complex surgical procedures [16]. When comparing Dexter and da Vinci, there are several aspects that must be learned with Dexter in order to complete the “Dexter learning curve”. Specifically, these are: only 2 instruments instead of 3 with da Vinci, different movement restrictions, each instrument has a separate patient cart (da Vinci only has one), different instruments, different foot pedal and linkage.

Complex surgical procedures are another point that should be discussed in the context of a new operating system. Compared to conventional laparoscopy, a robotic system can be used to perform more complex and surgically demanding operations in a safer and more targeted manner [17]. The main advantages here are functional (greater freedom of movement, an additional arm for better control and handling, better overview, less tremor due to greater stability) [4, 5]. In this respect, the switch from one operating system to another represents progress for surgery and thus also for patient care and the healthcare system as a whole [18, 19].

The initial uncertainties and perceived “setbacks,” such as longer operating times, can therefore be accepted without reservation. As such, enormous benefits can be seen at all levels once the learning curve has been completed [20, 21]. Such advantages become apparent on comparing conventional laparoscopy with the da Vinci system. In the present study and the interviews conducted, the establishment of a new robotic system (in addition to the existing da Vinci system) could not be considered equivalent to the latter in terms of the complexity of the procedures. Here, the already established system was named the leader for difficult and challenging operations. Nevertheless, the Dexter robot represents a niche for less advanced robotic systems, which then only cover a smaller spectrum, but may be more cost-effective and bridge the gap between conventional laparoscopy and the da Vinci system. This would permit full utilization of the advantages of the robot in a wide range of moderately demanding operations; the advantages include uncomplicated docking and undocking, and thus minimization of time loss.

A key advantage of the Dexter robot is the fact that, unlike the da Vinci system, the surgeon operates from a sterile console and can therefore assist at the operating table at any time, whether in an emergency situation or to support the assistant [22, 23]. One could even go a step further and perform Dexter operations with just one surgeon. There would no longer be a need for a medical assistant. Instead, operations could be performed with the sole assistance of an OR nurse or a student. This special concept could be marketed under a name such as “One-Surgeon Surgery (OSS)”. This approach would save resources for the clinic, which could then be used to compensate a part of the acquisition costs of the Dexter robot.

When a new operating system is introduced, it is important to prepare and train the surgical team, especially surgeons, optimally for the new conditions. Changes in handling, such as the arrangement of the power pedals, require practice and familiarization. In the present study, the interviewees described a certain degree of uncertainty and skepticism towards the new system, which can primarily be explained by a lack of experience. This hurdle can be overcome by simulator training as an essential and, ideally, mandatory tool for surgeons to become familiar with the new system. We recommend a mandatory training curriculum as part of the implementation of any new robotic system. Both surgeons and surgical assistants should be trained. Such a curriculum could be structured as follows: 30 h of training on the virtual reality simulator (5 h of training for the surgical assistants), various modules that can only be unlocked step by step. Subsequently, training in the operating room directly on the new robot should be recommended, as well as the presence of the manufacturer during the first 10 procedures performed by each surgeon to clarify any questions or resolve any uncertainties. Ackermann et al. performed a study concerning advanced surgical techniques and the factors influencing their surgical performance. The training scores of persons attending 16 standardized training courses of the German Working Group for Gynecological Endoscopy (AGE e.V.), individual characteristics, and the results of psychomotor tests of three-dimensional imagination and hand–eye coordination were correlated. The strongest correlation was noted for good surgical performance and learning success, and the absolute number of performed laparoscopic surgeries. The authors conclude that the possibilities of surgical training should be improved, promoted, and made accessible to a maximum number of surgical trainees because individual learning curves can be overcome even by less skilled or talented surgeons [24]. In this respect, simulator training should be an integral part of everyday clinical practice and go hand in hand with the implementation of a new surgical system. Surgeons should complete an appropriate training curriculum on the simulator in order to optimize their surgical skills for use on patients [7, 24].

A systematic review and meta-analysis performed by Alaker et al. confirmed this statement by comparing virtual reality simulation in laparoscopic abdominal surgery compared to other simulation models and no training. The authors registered an improvement in surgical performance and shorter operating times as a result of simulator training [25].

In the current report, participants of the surgery team who were directly involved in the delicate phase of implementing a new robotic system were interviewed in order to gauge the mood and identify the potential advantages and disadvantages of the new robotic system. Although the latter offers many functional advantages, which were also recognized as such, the interviewees preferred the established and familiar system. It should be noted that the learning curve for the new system was far from complete at the time of the interviews, so a final assessment is still pending. There is no question that the Dexter robot is a good surgical system with many advantages, and the updates from version 1 to version 2 have already brought about many technical improvements. It should also be noted that there were many skeptics and critics when the da Vinci was first implemented. In this respect, the Dexter robot can be described as a robust operating system that will find its niche in everyday clinical practice.

## Conclusion

The implementation of a new surgical system poses enormous challenges for the entire surgical team. Initial uncertainties in handling the system can be overcome once the learning curve has been completed. Surgeons should be offered simulator training as a standard measure in order to gain more confidence in using the operating system in advance, and to alleviate ethical concerns regarding patient safety. A new operating system is a technical and economic advance that will benefit both the clinic and patients, and thus the entire healthcare sector. In conclusion, new operating systems should be given a fair chance as they often offer many advantages once the learning curve has been completed. Subsequently they contribute to scientific, economic, and clinical progress.

## Supplementary Information

Below is the link to the electronic supplementary material.

Supplementary Material 1: Interview questionnaires (surgeons, OR nurses, anesthesiologists)

## Data Availability

The datasets analyzed for the current review are available from the corresponding author on reasonable request.

## Abbreviations

OR nurses: Operating-room nurses
