# Supplementary material for: Challenges and opportunities in the implementation of a new robotic system – a semi-structured interview study with a surgical team
Source: J Robot Surg. 2026 Jan 10;20(1):164. doi: 10.1007/s11701-025-03097-4 (PMC12789171; doi:10.1007/s11701-025-03097-4)
Supplement: Supplementary file 1 — Supplementary Material 1: Interview questionnaires (surgeons, OR nurses, anesthesiologists) [file 11701_2025_3097_MOESM1_ESM.docx]

**Interviews**

Comparative study of the da Vinci and the Dexter robot

Guideline

1. Personal questions about the interviewee

- Occupation
- Age
- Sex
- Specialization
- Professional experience in years
- Direct contact with patients (yes/no)
- Function (Doctor, Nurse, Staff member)
- Number of years at the UKSH Clinic

1. Evaluative questions concerning da Vinci and Dexter
2. Questions addressing economic motives

**Anesthesia**:

1. **Personal questions about the interviewee**
2. **Evaluative questions concerning da Vinci and Dexter**

- With regard to communication between colleagues – is it very different when operating with Dexter? If yes, how?
- With regard to anesthesia – do you see any advantages or disadvantages for one of the two robots?
- Anesthesia, induction, conclusion, positioning
- Do you see any changes in the anesthesia procedure?
- Is access to the patient limited by any of the two robot systems (spatially)?
- Is the procedure of docking to one of the two robots easier/more difficult and therefore faster/slower?
- Which of the two robots is the surgeon able to handle with greater confidence? Do you think that, after sufficient training, both robots can be used equivalently?
- The da Vinci robot is much larger and more cumbersome and needs more space in the operating room. Do you experience this as a hindrance in surgical situations?
- Is operating with Dexter now better established than it was at the time of the survey? (the time period of data collection is from November 2022 to April 2023)
- Which robot do you generally think is the better one?

1. **Questions addressing economic motives and the ethical aspect**

- Would you give preference to one of the two robots if you yourself or a friend of yours were to be operated on?
- Do you feel that patients enter the operating room with their usual “calmness” when they are operated on with the “new, unpracticed” Dexter compared to the da Vinci robot?
- Do you think the patient’s wellbeing is endangered when the operation is conducted with Dexter?
- Has the error rate proven to be higher with one of the two robots?
- Is the atmosphere in the operating room more tense when the operation is to be conducted with Dexter? Do more incidents occur? Or is the atmosphere more relaxed when the operation is conducted with da Vinci?
- Is the duration of the operation shorter with Dexter or da Vinci?
- Do you think a shorter duration of operation is an expression of the Clinic pursuing its economic interests?

**Nursing:**

1. **Personal questions about the interviewee**
2. **Evaluative questions concerning da Vinci and Dexter**

- Is the procedure of docking to one of the two robots easier and therefore faster?
- Would you give preference to one of the two robots if you yourself or a friend of yours were to be operated on?
- Do you think that, given sufficient training, Dexter will be equivalent to da Vinci?
- Is one of the two robots easier to handle? If yes which one?
- Is operating with Dexter now better established than it was at the time of the survey? (data collection from November 2022 to April 2023).
- Does positioning differ with the two robots? If yes, is one better than the other?
- The da Vinci robot is much larger, more cumbersome, and needs more space in the operating room. Do you see this as a hindrance in surgical situations, especially when you have been appointed as a standby or backup?
- At the Dexter robot the surgeon no longer sits in a closed console; he/she operates in an open console. Does this kind of communication simplify the surgical situation and atmosphere?
- Which robot do you generally prefer?
- In terms of time coordination: is it laborious to transfer the Dexter from one operating room to the other?
- Does such a new implementation (of a new robot) signify a major change in surgical management?
- What are the risks/challenges of the new robot?
- What do you think are the opportunities?
- Is the new robot a major change for surgical nurses, possibly also associated with stress and a higher error rate?

1. **Questions addressing economic motives and the ethical aspect**

- Do you think that patients enter the operating room with the same calmness when they are to be operated on with the “new and unpracticed” Dexter? Compared to da Vinci?
- Is the atmosphere in the operating room more tense or is it the same when the operation is to be conducted with Dexter? Or is the atmosphere more relaxed when the operation is conducted with da Vinci?
- Do more incidents occur with the Dexter robot?
- Which of the two robots does the surgeon handle with greater certainty? Or do you see no difference?
- Do you think the patient’s wellbeing is seriously endangered when the operation is performed with Dexter?
- Do you think that, given sufficient training, both robots can be used equivalently?
- Is the error rate higher with one of the two robots?
- Is the duration of the operation shorter with Dexter or da Vinci?
- Is patient safety ensured to a greater extent with one of the two robots?

If yes, do you think it is ethically justifiable to operate a patient with the less safe robot?

**Surgeons:**

1. **Personal questions about the interviewee**
2. **Evaluative questions about Dexter and da Vinci**

- Do you think that, given sufficient training, Dexter will be equivalent to da Vinci?
- Does Dexter offer advantages compared to da Vinci? If yes, what are they?
- Possible suggestions: surgical, degrees of freedom, communication, docking.
- Is the surgeon’s ergonomic position possibly advantageous with Dexter? Possibly also in preventing physical complaints for surgeons?
- With Dexter the surgeon is sterile and can, at any time, switch to the laparoscopic mode of surgery. Do you think robot-assisted surgery will, in the long term, be superior to conventional laparoscopic surgery and the aspect of sterility will therefore become secondary?
- Or do you think the surgeon will never be able to rely on the robot 100% even in the future and therefore a rapid switch (20 seconds for Dexter) from the robotic-assisted surgery to laparoscopic surgery will be advantageous?
- Is one of the robots easier to handle? If yes which one?
- Do you think that, given sufficient training, Dexter will be equivalent to da Vinci?
- Is operating with Dexter now better established than it was at the time of the survey? (data collection from November 2022 to April 2023).
- Does the procedure of positioning differ for the two robots? If yes, is one better than the other?
- Is the procedure of docking to one of the two robots easier and therefore faster?
- *Open question: Which robot do you generally prefer?*

1. **Questions addressing economic motives and the ethical aspect**

- Do you feel less confident with Dexter and are therefore more stressed out in the surgical situation? If yes, do you think the patient’s wellbeing is seriously endangered (e.g. a software crash while operating with Dexter).
- Is the duration of the operation shorter with Dexter or with da Vinci? Could, for instance, the risk of infection be reduced with a shorter operating time?
- Could a shorter operating time also mean a saving of costs? (because more operations could then be performed in the same time period).
- Is patient safety ensured to a greater extent with one of the two robots?

If yes, do you think it is ethically justifiable to operate on a patient with the less safe robot/less confident surgeon?

- Do you think your medical decisions sometimes take a secondary position due to the medical guidelines of the Clinic?
  - If yes, how frequently does that happen?
  - If yes, does it also apply to the robot systems? Are you encouraged (by the owners of the Clinic) to use Dexter in order to ensure economic advantages for the Clinic although the patient might be endangered by it?
- Would you – by the current state of the art – have a friend operated on with Dexter or with da Vinci?
- Is the error rate higher with one of the two robots?
- Do more incidents occur with Dexter?

**Free text**

- Is there anything else you consider important that has not been addressed here?
- If you were to wish one of the robots for yourself, which one would it be?
- Do you have any further remarks?
